# Perceptions of Spiritual Dryness in Iran During the COVID-19 Pandemic

**DOI:** 10.1007/s10943-021-01360-0

**Published:** 2021-07-29

**Authors:** Arndt Büssing, Sara Hamideh Kerdar, Mohammad Esmaeil Akbari, Maryam Rassouli

**Affiliations:** 1 Faculty of Health, Witten/Herdecke University, Gerhard-Kienle-Weg 4, 59313 Herdecke, Germany; 2 Philosophical-Theological Academy, IUNCTUS - Competence Center for Christian Spirituality, Münster, Germany; 3 Faculty of Health, Witten/Herdecke University, Herdecke, Germany; 4 Cancer Research Center, Shahid Beheshti University of Medical Sciences, Tehran, Iran

**Keywords:** Spiritual dryness, Spiritual crisis, Spirituality, Validatio, Spiritual dryness scale, Muslims, Iran, COVID-19 pandemic

## Abstract

This study addresses perceptions of spiritual dryness (a specific form of spiritual struggle) during the COVID-19 pandemic among Iranian Muslims (*n* = 362), and how these perceptions can be predicted. Spiritual dryness was perceived often to regularly by 27% and occasionally by 35%. Regression models revealed that the best predictors of spiritual dryness (SDS-7) were usage of mood-enhancing medications, loneliness/social isolation and praying as positive predictors, and being restricted in daily life concerns as negative predictor. The pandemic challenges mental stability of people worldwide and may also challenge trust in God. Reliable and humble support of people experiencing these phases is required.

## Introduction

Studies have shown that people's attention to spirituality may increase in times of crisis (Rahnama et al., 2012)*.* One of the most important examples of such crises in the twenty-first century is the COVID-19 pandemic, which has affected societies in different ways. From the beginning of this pandemic, the moments of fear, pain, loneliness and the sense of imminent death have been experienced by patients and all those who are at risk, as well as anyone who perceives its dangers and adverse outcomes (Büntzel et al., 2020; Büssing et al., 2020a, 2020b; Heidari et al., 2020).

Depending on the influence of spirituality and religion on each society, spirituality is considered as a resource to cope with the adverse impact of diseases on people's health (Weber & Pargament, 2014). In addition, religions provide a frame of reference for understanding the experience of suffering, pain and finality (Daaleman & VandeCreek, 2000). Although both terms are often used interchangeably, these are conceptually not identical. In relatively secular societies, spirituality is regarded as a more comprehensive and “individual” opposite of institutional religiosity with its specific doctrines, belief systems and rituals (Büssing, 2012, 2019). *Religion* is understood as “an institutional and culturally determined approach which organizes the collective experiences of people (faith) into a closed system of beliefs and practices (‘form’)” (Büssing, 2012), while the more open term *spirituality* “refers to the individual experiences of the Sacred” or to an individual search for meaning in life along with subsequent values, ethics, rituals and practices (Büssing, 2012, 2019). The broader and more general concept*, spirituality*, is defined based on religion and culture, affecting people’s perception of health and illness (Rahnama et al., 2012)*.* A qualitative study conducted in Iran proposes, “Spirituality is the sublime aspect of human existence bestowed on all humans in order for them to traverse the path of transcendence that is closeness to God (Allah).” (Memaryan et al., 2016). The definition is similar to the previous definitions of this concept, both in its main part (transcendence) and in incorporating a God-centered view of spirituality within the context of an Islamic society (Alzahrani et al., 2016).

In this regard, one may differentiate positive and negative religious coping strategies, with different outcomes on mental health indicators (Weber & Pargament, 2014). Times of crisis may challenge one’s religious convictions and faith while facing negative experiences; one’s faith may also be utilized as a resource to cope, when positive experiences take place (Weber & Pargament, 2014). This may also result in spiritual consolidation or “awakening” and coherence, which is a sense of harmony, connection and meaningfulness (Lips‐Wiersma, 2002; Nemati et al., 2017).

Apart from these rather positive aspects, there might be also times of religious affliction (“spiritual struggles”) which are associated with a sense of disharmony, disconnection, loss of meaning and purpose in life (Smucker, 1996) as well as leading to passive reactions such as despair, hopelessness, depression and ultimately indifference (Weber & Pargament, 2014). In fact, religious people may perceive spiritual struggles that either refer to the faith community, to religious doctrines and beliefs, or to God; these struggles may affect their wellbeing (Exline & Rose, 2013; Exline et al., 2014, 2015; Stauner et al., 2016).

Spiritual struggles are in general an important and diverse field of research, as there are associations between childhood trauma and spiritual struggles in adulthood, Czech participants particularly face demonic struggles (Janů et al., 2020), while alcohol problems were somewhat associated with all types of spiritual struggles, too (Stauner et al., 2019). However, “only moral struggle predicted alcohol problems moderately and independently of religiousness, distress, gender, and non/white ethnicity,” as stated by Stauner et al., 2019. Moral injuries were in fact relevant triggers of these general spiritual struggles (Currier et al., 2019), which are associated with suicidal behaviors, too (Currier et al., 2018).

However, not all of these struggles are the indicators of psychopathologic disorder and do not necessarily require a psychological/psychiatric intervention. Some of these spiritual struggles refer to extrinsic factors (i.e., the religious community and their ethics and beliefs), to intrinsic causes (i.e., loss of meaning in life), and some to the relation with God, who is perceived as distant and non-responding (Exline et al. 2013; Büssing et al., 2013, 2020b, 2021c). Therefore, the outcomes might be different, too.

Global crises such as the COVID-19 pandemic may thus challenge a person’s religious trust and faith in God who might be regarded as less interested in man’s concerns in difficulties in life (Büssing et al., 2021a). In a recent study, Iranian nurses stressed the importance of providing spiritual care in the pandemic period and stated that the lack of this type of care causes spiritual distress in patients. According to the nurses, spiritual care can help to stabilize patients’ emotional situation during the COVID-19 pandemic and may allow them to “continue living” (Rezaee et al., 2020).

It is assumed that one way to cope with the social and individual overcome of the COVID-19 pandemic is strengthening religious faith, because developing a stronger bond with God would reduce or eliminate distress, anxiety and stress and increase hope and peace (Kowalczyk et al., 2020; Roman et al., 2020). This applies the ability to utilize persons’ faith in terms of positive religious coping. Religions, in general, and religious communities, specifically, may further contribute to promote health politics, social and health behaviors, meaning finding and coping strategies (Barmania & Reiss, 2020). However, depending on the cultural and religious background, people may or may not utilize their faith to cope with the COVID-19 pandemic. For example, in German patients with cancer, interest in spirituality during the pandemic was rather low, while their perception of nature as a source of consolidation, and quiet times of reflection were of stronger interest (Büssing et al., 2020d). That faith is a strong hold to them was stated by only 31%. Moreover, during the course of the pandemic, tumor patients’ religious trust and prayer practices were significantly higher directly after the first lockdown compared to the beginning of the second wave of the pandemic (Büssing et al., 2021a). This could mean that the stress related to the pandemic and its burden may have an impact on their spiritual attitudes and practices.

Iran is one of those countries with relatively high death rate due to the COVID-19 infections (Rassouli et al, 2020). Here, 98% of people are Muslims (Rassouli & Sajjadi, 2016)*,* and their religious belief is considered as an important source for coping with stressors. In fact, spirituality is an essential element in Iranians’ lives and is rooted in their culture and history (Nemati et al., 2017). However, some Iranians, based on their spiritual beliefs, may nevertheless have contradictory opinions on life events and God (Nemati et al., 2017). Some may consider God responsible for the negative life events and regard themselves as victims (Exline et al., 2014), interpreting these events as punishment and injustice from God. Moreover, in Western societies, negative life events (i.e., illness) are interpreted as not only the interruption of life, failure or punishment, but also as a challenge or as a valuable motive for growth (Büssing & Fischer, 2009; Büssing & Surzykiewicz, 2015; Degner et al., 2003). With respect to Islam, it should be noted that *justice* is one of the principles and *disbelief* is considered as heresy. Yet, this might be a misconception of “divine justice,” considering justice as laws that God is obliged to follow (Rokni, 2008). However, according to Islam, God is the *source* of justice and everything He does is justice; therefore, He has set a proper path for every creature to reach perfection (Motahhari, 2013). In this regard, studies have shown that spirituality, through creating a positive attitude, may foster outcomes such as a sense of worthiness and peace, acceptance of current situation and patience (Soleimani et al., 2016). Thus, in such situations, very religious people may regard these apparently devastating crises as the best blessings in life (Nemati et al., 2017)*,* while others struggle with their faith because they cannot share this perspective.

The times of crisis are also the times of religious struggles and insecurities (Exline et al., 2014). In fact, spiritual struggles, doubts about God and His closeness, as well as the perceived distance toward God, who seems not to care about mans’ concerns, are everlasting experiences and have been articulated for millennia (Büssing & Dienberg, 2019). This may also be true during the COVID-19 pandemic. Therefore, identifying the barriers such as spiritual dryness and its predictors is of great importance.

Spiritual dryness refers to the perception that God is distant, regardless of our efforts to get close to Him, that one’s prayers go unanswered, as well as the feeling of being completely abandoned by God, the feelings of being “spiritually empty” and not being able to give any more (Büssing et al., 2013, 2020b, 2021c). Similar concepts described in the mystic literature are “dark night of the soul,” “spiritual desolation” or “desert experience,” which nevertheless may be different from a theoretical point of view (Büssing et al., 2020a). These concepts refer primarily to spiritual struggles without the assumption of an underlying psychopathology. Nevertheless, in palliative medicine, there is an important topic, which may be in part related to spiritual dryness, termed “spiritual distress” (Puchalski et al., 2018). It refers to any impairment in the experience of meaningful life that should normally be acquired through communication with self, others, the world or a superior power (Caldeira et al., 2017). Spiritual distress refers to both, depressive symptoms (i.e., depression, anxiety, hopelessness, disturbance of sleep) and spiritual distress (i.e., feeling of being abandoned by God, religious doubt, loss of faith). Spiritual distress is thus a broad concept and does not necessarily depend on a particular religion or specific beliefs. Both religious and non-religious individuals may experience such spiritual distress (Nolan et al., 2020). However, “spiritual distress” is usually seen as disease related or triggered by the impact of disease and its prognosis, while spiritual dryness is also and foremost experienced by “healthy” persons (Büssing et al., 2020a).

The underlying causes of spiritual dryness can be differentiated as extrinsic and intrinsic (not in terms of exclusive states, but polarities) (Büssing et al., 2020b). Some attributed these perceptions to temptations of evil spirits or hint that spiritual life needs to be changed, or to the need to let go of all materialistic concerns, or to an “inertia of the heart” in terms of a boredom or negligence (Büssing et al., 2020a). Modern scholars discuss that spiritual dryness can also be seen in the context of depressive states (Bäumer & Plattig, 2008; Durà-Vilà, 2017; May, 2009; Ott, 1982). In several studies on Christian pastoral workers, religious brothers and sisters, and lay people, spiritual dryness was experienced by 12 to 14% often to regularly (Büssing & Dienberg, 2019; Büssing et al., 2016, 2017a, 2017b, 2018a, 2018b, 2018c) and by 16% in Seventh-day Adventists (Büssing et al., 2021c). The underlying causes were heterogeneous, ranging from the lack of ability to perceive the Sacred in life and low coherence in life to emotional exhaustion and depressive symptoms (Büssing et al., 2016, 2017a, 2017b). Excessive Spiritual demands, the low perception of the Sacred in daily life concerns, difficulties in prayer life as well as higher influence of emotional exhaustion and low wellbeing were reported, too (Büssing et al., 2021c). Depending on the cohort, the predictors of spiritual dryness perceptions may vary. The experience of spiritual dryness is not necessarily a matter of “spiritual weakness,” but is an existential experience that requires support, as it may be a turning point toward either spiritual development and the consolidation of faith, or spiritual desolation and loss of faith (or disinterest) (Büssing & Dienberg, 2019; Martini & Bader, 2005).

This study aims to find out whether and how the Muslims in Iran would perceive the phases of spiritual dryness, and how these are related to the indicators of spirituality and wellbeing as reported in other studies (Büssing et al., 2016, 2017a, 2017b, 2018a, 2018b, 2018c, 2021c). The perception of awe/gratitude (as a perceptive aspect of spirituality) and religious practices (particularly the spontaneous private prayers rather than the frequency of obligatory religious practices and prayers) were both negatively related to spiritual dryness in German pastoral workers (Büssing et al., 2017b); thus, similar associations can be assumed among Iranian Muslims, too. Furthermore, it is suspected that low wellbeing, low life satisfaction and a variety of stressors related to the COVID-19 pandemic (e.g., daily life restrictions, the feelings of being stressed/under pressure, of being fearful/insecure, lonely/socially isolated, as well as the financial burden) will have an impact on the perception of spiritual dryness. The impact of health behaviors on spiritual dryness has never been investigated and is part of this analysis.

## Materials and Methods

### Study Participants

The participants from Iran took part in an anonymous online survey with standardized measures through convenience sampling method from December 2020 to February 2021. The study was approved by the Ethic Committee of Cancer Research Center, Shahid Beheshti University of Medical Sciences (IR.SBMU.CRC.Rec.1399.027). The invitations to participate, along with a short explanation about the study and its purpose, were posted in several social media platforms as well as different groups on WhatsApp and Telegram and forwarded in various Iranian networks with the request to forward it with the purpose of snowball sampling. Upon willingness to participate in the study, the participants signed an online consent form before accessing the questionnaire. Neither the concrete identifying personal details nor the IP addresses were recorded to guarantee anonymity. The inclusion criteria were being a Farsi speaker and over 18 years of age. The sample also included seven Muslims with Iranian nationality living in other countries.

### Measures

The online questionnaire consisted of basic sociodemographic questions regarding gender, age, nationality and religious affiliation. All the measures which were used in the Farsi version, will be described below. The translation process started with the first translation from German/English language to Farsi by a bilingual researcher. Afterward, it was checked and adjusted by an additional bilingual researcher. After back translation into English, the inconsistencies were addressed. After the Farsi version was prepared, the tool was psychometrically evaluated by measuring the content validity and performing cognitive assessment. In order to evaluate the content validity, seven experts in nursing, psychology and psychometrics were asked to express their opinions on the compatibility of the items with the cultural circumstances of Iran. Then the cognitive assessment of the tool was performed using the opinions of 10 participants meeting the inclusion criteria (individuals over 18, fluent in Farsi) in order to determine the simplicity and the comprehensibility of the items.

### Spiritual Dryness

The perception of “spiritual dryness” was assessed with the Spiritual Dryness Scale (SDS). It primarily uses six items with good internal consistency (Cronbach’s *α* = 0.87) (Büssing et al., 2013). Specific statements refer to the feelings that God is distant, that one’s prayers go unanswered, as well as the feeling of being “spiritually empty” or not able to give any more (in terms of spiritual exhaustion) and being abandoned by God. The items of this instrument were formulated in order to fit into the daily life experiences of religious individuals. For this analysis, we added an additional item, which asks for a “deep longing for God” (item SDS0). The response options on a Likert scale were not at all (1), rarely (2), occasionally (3), fairly often (4) and regularly (5). The SDS scores are the mean scores and represent the perceived lack/shortage.

The instrument uses three further items to address whether one has “found ways to deal with these feelings” in terms of coping strategies, and behaviors and perceptions when these phases would overcome: “These feelings inspire me to help others more.” and “After these phases, I experience greater spiritual serenity and depth.” The scoring is the same as the above mentioned.

### Awe and Gratitiude

Awe/Gratitude as an indicator of experiential spirituality was measured with the 7-item Gratitude/Awe questionnaire (GrAw-7) (Büssing et al., 2018c) which has good psychometric properties (Cronbach’s *α* = 0.82). This measure has a clear focus on the experiential aspects of being moved and touched by certain moments and or distinct locations, nature-related reactions of pausing daily activities and the subsequent feelings of awe and gratitude. The specific items are, “I stop and then think of so many things for which I'm really grateful,” “I stop and am captivated by the beauty of nature,” “I pause and stay spellbound at the moment” and “In certain places, I become very quiet and devout.” All the items were scored on a 4-point scale: 0, never; 1, seldom; 2, often; and 3, regularly, based on a 100% scale. The resulting mean values thus range from 0 to 100.

### Faith as a Strong Hold

To measure a more specific form of religiosity, we added item A37 from the Reliance on God’s Help scale (Büssing et al., 2015), which asks whether faith is a strong hold in difficult times. Agreement or disagreement was scored on a 3-point scale: 0, disagreement; 2, indifference; and 3, agreement. This item was used as a differentiating variable to assess intrinsic religiosity in terms of attitude.

### Praying and Meditation

To assess the behavioral and ritual aspects of religiosity, the frequency of praying and meditation was differentiated, ranging from never (0), at least once per month (1), at least once per week (2), to at least once per day (3). Praying was further differentiated as an obligation, i.e., performing the obligatory prayers (Salat), or as a spontaneous reaction, i.e., private prayer (Du’a). The frequency of both was scored as never (0), seldom (1), often (2) and regularly (3).

### Wellbeing

Wellbeing was assessed with the 5-item WHO-Five Well-being Index (WHO-5) (Bech et al., 2003). The representative items are “I have felt cheerful and in good spirits” or “My daily life has been filled with things that interest me.” The intensity of feelings refers to the last two weeks and was scored with a 6-step grading scale ranging from at no time (0) to all the time (5). Here, the reported WHO-5 sum scores refer to a 100% level [0–100], in which the scores < 50 are the indicators of reduced wellbeing (Toppet al., 2015).

### Life Satisfaction

Life satisfaction was measured using the Brief Multidimensional Life Satisfaction Scale (BMLSS) (Büssing et al., 2009). The items of the BMLSS address intrinsic (oneself, life in general), social (friendships, family life), external (work situation, the place of residence) and prospective dimensions (financial situation, future prospects) of life satisfaction as a multifaceted construct. The internal consistency of the instrument was found to be good in the validation study (Cronbach’s *α* = 0.87). In this study, the 10-item version was employed that included satisfaction with the health situation and abilities to deal with daily life concerns (BMLSS-10).

### Perception of Burden

The perceived restrictions of daily life (pressure/stress, anxiety/insecurity, loneliness/social isolation and the restrictions of financial–economic situation related to the COVID-19 pandemic) were measured with five numeric rating scales (NRS), ranging from 0 (not at all) to 100 (very strong) as described (Büssing et al., 2020c). These five variables can be combined in a factor termed “Stressors” (5NRS) with good internal consistency in this sample (Cronbach’s *α*  = 0.80).

### Frequency of Health Behaviors

Health behaviors such as physical/sport activities, walking outside in the nature, and taking mood-enhancing medications (in terms of antidepressants or anti-anxiety medications, but not in terms of illicit drugs) were measured with a 4-grade scale (never, at least once per month, at least once per week, at least once per day) as described (Büssing et al., 2020c).

### Statistical Analysis

Descriptive statistics, the analyses of variance (ANOVA), first-order correlations (Spearman rho), regression analyses as well as internal consistency (Cronbach’s coefficient α) and factor analyses (principal component analysis using Varimax rotation with Kaiser’s normalization) were computed with SPSS 23.0.

To analyze the predictors of spiritual dryness, we performed three regression models to assess which independent variables would predict SDS scores as a dependent variable: (1) the indicators of spirituality, (2) the indicators of the quality of life and (3) health behaviors such as the intake of mood-enhancing medications.

Given the exploratory character of this study, the significance level of ANOVA and correlation analyses were set at *p* < 0.01. With respect to classifying the strength of the observed correlations, we regarded *r* > 0.5 as a strong correlation, an *r* between 0.3 and 0.5 as a moderate correlation, an r between 0.2 and 0.3 as a weak correlation and *r* < 0.2 as negligible or no correlation.

## Results

### Description of the Sample

The sample consists of 362 participants from Iran (53% men; 45% women; and 2% “other”) with a mean age of 39.5 ± 17.3. All were Muslims, most were living in a family household (Table 1). Deep longing for God/Allah was perceived often to regularly by 57%, sometimes by 27% and not at all or seldom only by 17%. Private prayers were practiced more often (6%, never) than obligatory prayers (20%, never). Within the sample, life satisfaction (BMLSS-10) scores were in the upper mid-range and wellbeing scores (WHO-5) in the mid-range, while the perceived burden scores (5NRS) are in the lower mid-range. In addition, the perceptions of Awe/Gratitude were in the mid-range. With respect to the participants’ health behavior (Table 1), usage of mood-enhancing medications was quite common, 20% at a daily level; 25% at least once per week; 21% at least once per month; and only 34% never. Sporting activities were practiced by 62% at least once per week or at a daily level, and walking outside by 57% at least once per week or at a daily level. Similarly, meditation (the style and the content were not specified in the questionnaire) was practiced at least once per week or at a daily level by 50% and praying by 71% (Table 1).

**Table 1:** Description of 362 Muslim participants from Iran

	*n*	%	Mean ± SD
*Sociodemographic data*			
Gender	362		
Male	193	53	
Female	162	45	
Other	7	2	
Age (years)	302		39.5 ± 17.3
Living situation	362		
Family household	290	80	
Single	59	16	
other	13	4	
Area of work	362		
Administration/management	24	7	
Economy	39	11	
Medicine	90	25	
Psychology	39	11	
Education	43	12	
Craft/trade	26	7	
Other	101	27	
*Indicators of spirituality*			
Deep longing for God [0–4]	351		2.62 ± 1.13
Not at all/seldom	58	17	
Sometimes	93	27	
Often/regularly	200	57	
Frequency of prayer [0–3]	330		2.0 ± 0.9
Frequency of private prayers (Du’a) [0–3]	332		1.8 ± 0.8
Never	19	6	
Seldom	95	29	
Often	151	46	
regularly	67	20	
Frequency of obligatory prayers (Salat) [0–3]	333		1.7 ± 1.1
Never	65	20	
Seldom	72	22	
Often	98	29	
Regularly	98	29	
*Indicators of wellbeing*			
Life satisfaction [0–100]	362		56.3 ± 16.6
Wellbeing [0–100]	362		55.2 ± 16.0
Awe/Gratitude [0–100]	360		57.4 ± 15.1
*Perceived burden due to the pandemic*			
Restrictions of daily life [0–100]	362		40.2 ± 27.8
Pressure/feeling stressed [0–100]	362		40.7 ± 28.2
Anxiety/insecurity [0–100]	361		38.9 ± 28.0
Loneliness/social isolation [0–100]	360		37.8 ± 29.0
Financial–economic situation [0–100]	362		36.1 ± 28.8
Perceived Burden (5NRS) [0–100]	362		38.7 ± 20.9
*Health behaviors*			
Mood-enhancing medications [0–3]	335		1.3 ± 1.1
Sporting activities [0–3]	334		1.7 ± 0.9
Go out for walking [0–3]	329		1.7 ± 0.9
Meditation [0–3]	331		1.4 ± 1.1

Not all participants responded to all items

### Perception of Spiritual Dryness

Among 362 participants, six did not respond to the Spiritual Dryness Scale (1.7% non-responder). Here, the indicators of spiritual dryness were perceived with varying intensities by the participants (Table 2). The general experience of spiritual dryness was perceived often to regularly by 27%, occasionally by 35%, rarely by 23% and not at all by 15%. That prayers go unanswered was experienced often to regularly by 28%, distance from God often to regularly by 16%, while the feeling of being abandoned by God was experienced often to regularly by 24%. Being “spiritually empty” was experienced often to regularly by 28% and the perception of not being able to emotionally “give” any more by 22%.

**Table 2:** Frequency of spiritual dryness perceptions (%)

	Not at all	Seldom	Sometimes	Often	Regularly
*Item phrasings*					
I have the feeling that God is distant from me, regardless of my efforts to draw close to him	28	32	24	12	4
I have the feeling that my prayers go unanswered	21	19	32	19	9
I have the feeling that God has abandoned me completely	30	20	26	18	6
I experience times of “spiritual dryness”	15	23	35	23	4
I have the feeling that I am “spiritually empty”	19	24	29	19	9
I know the feeling of not being able to give any more	19	29	31	14	8
*Marker Items when these feelings were experienced*					
I have found ways to deal with these feelings	8	20	30	27	15
These feelings inspire me all the more to help others	6	16	31	31	16
After these phases I experience a greater spiritual serenity and depth	19	20	25	26	11

Additionally, 42% of the participants stated that they had often to regularly found ways to cope with these phases; 30% occasionally; and 28% seldom or almost never (Table 2). When these phases were overcome, 47% stated that these feelings of spiritual dryness had often to regularly inspired them to help others more, while 37% had often to regularly experienced greater spiritual serenity and depth.

Principal component analysis (Varimax rotation) confirms that the 6-item Spiritual Dryness Scale (SDS) can be combined to a single-factor structure in Muslim cohorts too, which explains 50% of variance and has a good internal consistency (Cronbach’s *α* = 0.79) (Table 3).

**Table 3:** Mean values, reliability and factor analysis of the Spiritual Dryness Scale (SDS)

	Mean ± SD [0–4]	Corrected item—scale correlation	α if item deleted (*α* = .793)	Factor loading
*Item phrasings*				
I experience times of “spiritual dryness”	1.79 ± 1.09	.633	.742	.783
I have the feeling that I am “spiritually empty”	1.75 ± 1.22	.619	.743	.779
I have the feeling that God has abandoned me completely	1.50 ± 1.26	.612	.745	.764
I have the feeling that my prayers go unanswered	1.75 ± 1.22	.585	.752	.736
I know the feeling of not being able to give any more	1.63 ± 1.16	.426	.788	.573
I have the feeling that God is distant from me, regardless of my efforts to draw close to him	1.32 ± 1.12	.405	.792	.559
*Marker Items when these feelings were experienced*				
I have found ways to deal with these feelings	2.21 ± 1.17	–	–	–
These feelings inspire me all the more to help others	2.36 ± 1.10	–	–	–
After these phases I experience a greater spiritual serenity and depth	1.89 ± 1.28	–	–	–

Kaiser–Meyer–Olkin value of .84 and a significant Bartlett test (*p* < 0.0001). Extraction of the principal components (eigenvalue > 1); varimax rotation with Kaiser’s normalization; one factor explains 50% of variance

The SDS scores were the lowest in women and the highest in the very small subgroup of the people who stated their gender as “other” (Table 4). The differences are still significant (*F* = 15.8, *p* < 0.0001) without “other” gender participants, although the effect size was small (Cohen’s *d* = 0.42). Moreover, people who stated to have faith as a strong hold had lower SDS scores than people who cannot rely on this source (Table 4). Here, the effect size was small (Cohen’s *d* = 0.48). Age was marginally only related to SDS (*r* = 0.14, *p* = 0.014, Spearman rho).

**Table 4:** SDS scores differentiated for gender and faith as a strong hold (ANOVA)

	*N*	Spiritual Dryness Scale	
		Mean	SD
All participants	356	1.62	.83
*Gender*			
Men	188	1.77	.78
Women	161	1.43	.84
Other	7	2.26	.84
*F* value		10.1	
*p* value		< 0.0001	
Cohen’s *d* (men–women)		.42	
*Faith as strong hold*			
Does not apply	49	1.92	.63
Neither yes nor no	158	1.75	.68
Applies a lot	149	1.49	.97
*F* value		10.7	
*p* value		< 0.0001	
Cohen’s *d* (no–yes)		.48	

### Correlations Between Spiritual Dryness, Indicators of Spirituality, Wellbeing and Health Behaviors

Spiritual dryness is moderately negatively related to participants’ longing for God (Table 5). With respect to praying, it is weakly related to the frequency of private praying, but not significantly related to the frequency of obligatory prayers. Furthermore, awe/gratitude is not significantly related. However, SDS scores are moderately related to lower life satisfaction and only marginally with low wellbeing. Among the COVID-19 pandemic-related stressors, the perception of daily life restrictions was weakly negatively related, while loneliness/social isolation and financial border are weakly positively related to the SDS scores. Being under pressure/stress or feelings of fear and insecurity are not significantly related. The pandemic-related summarized stressor score (5NRS) is not significantly related to spiritual dryness (Table 5). With respect to health behaviors, the usage of mood-enhancing medications and meditation was moderately positively related to SDS scores, while sporting activities and going out for walking were only marginally related.

**Table 5:** Correlations between spiritual dryness and indicators of spirituality, wellbeing and health behaviors

	SDS score
*Indicators of spirituality*	
Frequency of praying	− .279**
Frequency private praying (Du’a)	− .214**
Frequency obligatory prayers (Salat)	.053
Awe/gratitude (GrAw-7)	− .065
*Indicators of wellbeing*	
Life satisfaction (BMLSS-10)	− .396**
Wellbeing (WHO-5)	− .180*
Perceived burden (5NRS)	.089
Daily life restrictions	− .266**
Under pressure/stressed	.007
Fearful/insecure	.107
Loneliness/social isolation	.253**
Financial burden	.203**
*Health behaviors*	
Mood-enhancing medications	.453**
Sporting activities	.122
Go out for walking	.168*
Meditation	.350**

**p < 0.001; *p < 0.01 (Spearman rho); moderate correlations are highlighted (bold)

### Predictors of Spiritual Dryness

Since several variables significantly related to spiritual dryness perceptions have been found, three regression analyses were performed to assess which independent variables would predict SDS scores as a dependent variable (Table 6). Gender (male or female) was tested first, but was less relevant, thus not used in the following three regression models (data not shown).

**Table 6:** Predictors of spiritual dryness analyzed in three steps

	Model 1: *R*^2^ = .26			Model 2: *R*^2^ = .38			Model 2: *R*^2^ = .45		
	Beta	*T*	*p*	Beta	*T*	*p*	Beta	*T*	*p*
Constant		15.561	< .0001		11.289	< .0001		9.405	< .0001
*Spirituality indicators*									
Faith as strong hold	− .176	− 3.483	.001	− .111	− 2.287	.023	− .111	− 2.387	.018
Longing for God	− .192	− 3.598	< .0001	− .120	− 2.359	.019	− .094	− 1.952	.052
Frequency of praying	− .148	− 2.794	.006	.237	4.931	< .0001	.135	2.764	.006
Frequency of meditation	.313	6.310	< .0001	− .114	− 2.323	.021	− .133	− 2.825	.005
*Quality of Life Indicators*									
Life satisfaction				− .168	− 3.225	.001	− .107	− 2.112	.036
Wellbeing				− .086	− 1.659	.098	− .060	− 1.227	.221
Restricted in daily life activities				− .226	− 4.317	< .0001	− .192	− 3.787	< .0001
Loneliness/social isolation				.173	3.208	.001	.146	2.830	.005
Burdening financial situation				.086	1.620	.106	.081	1.605	.109
*Health behaviors*									
Mood-enhancing medications							.223	4.353	< .0001

Data without “other” participants

In regression model 1, the indicators of spirituality were tested. These would predict 26% of SDS score variance, with the frequency of meditation as the best positive predictor, while praying, longing for God and faith as strong holds, are the negative predictors. In regression model 2, the quality of life indicators were added. Now, the predictors were the frequency of praying (which is a positive predictor now) and being restricted in daily life concerns (as a negative predictor), low life satisfaction and loneliness are further relevant predictors, while wellbeing and burdening financial situation had no significant relevance in this regression model. These analyzed variables would predict 38% of variance. Adding the intake of mood-enhancing medications in regression model 3, the best predictors are the usage of mood-enhancing medications (as a positive predictor) and being restricted in daily life concerns (still as a negative predictor). These variables would altogether explain 45% of SDS score variance.

### Praying and Meditation and Their Association with Spiritual Dryness, Restrictions, Life Satisfaction and Health Behaviors

It is important to underline that the frequencies of praying and meditation are not associated (*r* = 0.00). Those who meditate more often have lower perceptions of being restricted in their daily life activities (*r* =  − 0.26), but have marginally lower life satisfaction (*r* =  − 0.17), while they use mood-enhancing medications more often (*r* = 0.43). In contrast, those who pray more often have higher life satisfaction (*r* = 0.24), while there is no relevant association with perceptions of being restricted in daily life (*r* = 0.07); however, praying is not relevantly associated with the usage of mood-enhancing medications (*r* =  − 0.01). Neither of the spiritual practices is related to wellbeing (*r* = 0.052 and 0.053).

In the light of these findings, one can state that those who were praying at least once per day had the lowest spiritual dryness scores compared to those who prayed at least once per week. Additionally, they had the highest life satisfaction, while there were no significant differences for stressors (being restricted in daily life activities or loneliness/social isolation) and wellbeing (Table 7). In contrast, those who were never meditating had the lowest spiritual dryness scores, while those who meditated at least once per week or at a daily level had the highest spiritual dryness scores (indicating that meditation could have been used to cope). Furthermore, those who were never meditating had the highest perception of being restricted in daily life and the highest life satisfaction.

**Table 7:** Frequency of praying and meditation related to spiritual dryness, stressors, life satisfaction and wellbeing

	Spiritual dryness (SDS)	Restricted in daily life activities	Loneliness/social isolation	Life satisfaction (BMLSS-10)	Wellbeing (WHO-5)
All persons (*n* = 331)					
Mean	1.63	39.06	37.94	55.82	55.04
SD	0.81	27.23	28.79	16.23	15.68
*Praying*					
Never (*n* = 22)					
Mean	1.76	43.64	41.82	53.55	55.45
SD	0.72	24.79	26.12	21.13	17.99
At least once per month (*n* = 75)					
Mean	1.82	36.27	39.19	50.90	53.68
SD	0.63	27.35	27.09	13.95	15.62
At least once per week (*n* = 106)					
Mean	1.87	35.19	40.19	53.54	55.13
SD	0.74	25.83	28.69	13.55	13.41
At least once per day (*n* = 127)					
Mean	1.29	42.60	34.41	61.09	56.44
SD	0.86	28.46	30.62	17.30	17.52
*F* value	13.35	1.90	1.02	8.09	0.48
*p* value	< .0001	n.s	n.s	< .0001	n.s
*Meditation*					
Never (*n* = 96)					
Mean	1.18	51.56	40.00	60.17	53.25
SD	0.80	28.70	29.20	17.33	17.43
At least once per month (*n* = 69)					
Mean	1.68	38.41	34.64	53.42	54.32
SD	0.68	26.21	26.88	15.34	16.81
At least once per week (*n* = 99)					
Mean	1.85	30.81	38.16	55.61	56.75
SD	0.68	24.15	30.06	15.08	13.75
At least once per day (*n* = 67)					
Mean	1.89	34.03	38.06	52.36	55.82
SD	0.85	24.31	28.51	16.09	14.50
*F* value	16.95	11.57	0.47	3.92	0.91
*p* value	< .0001	< .0001	n.s	.009	n.s

## Discussion

Although the results of a comparative study (Asadzandi et al., 2020), conducted to compare the spiritual health behaviors of Iranians during the COVID-19 pandemic, showed that religious beliefs had a positive effect on the spiritual health of people, the findings of the current study underlined that the phases of spiritual dryness are nevertheless experienced by several participants. 27% of them perceived these phases often regularly. One may suggest the influence of the COVID-19 pandemic-related stressors on the participants’ SDS scores. However, among these cases, only a few were related to spiritual dryness. While the weak association between spiritual dryness and loneliness/social isolation is plausible from the conceptual point of view, which was found in another study, too (Büssing, et al., 2017a, 2017b), the perceptions of daily life restrictions due to the pandemic were, however, inversely related to spiritual dryness. This contra-intuitive association is so far difficult to explain, as a buffering effect seems unlikely.

Perceiving spiritual dryness is not necessarily a matter of “weakness in faith” or personal “failure,” but a complex spiritual and psychosocial development process (Büssing et al., 2020a). Some people, particularly when facing negative life events, feel that God has forgotten them, or that the event is a punishment from God, and they doubt God's power and think that He is unkind (Exline et al., 2015). In such a situation, it seems that there is either a lack of deep inner relationship with God or a less stable one (in terms of confidence and trust) (Büssing et al., 2020b, 2021c), and the fear of punishment and rejection by God is felt by the individual. Sometimes illnesses or difficult circumstances cause spiritual and religious crises in individuals that may reduce adaptation mechanisms and increase their anxiety levels (Mirhosseini 2020). In a qualitative study conducted in Iran, disappointment, the futility of praying and the feeling of being rejected by God were the emerging themes (Nemati et al., 2017). The participants revealed that they felt deprived of the divine attention and that they had lost their faith in spirituality. *S*ome of them believed that God was not noticing them or their prayers were not being answered. Unanswered prayers had led the participants to a feeling of being rejected by God. These perceptions are identical to the symptoms of spiritual dryness (Büssing et al., 2013), as reported in this study, too. The participants who reached this point appeared to have a defensive reaction to their spiritual beliefs that, in the most extreme cases, emerged as the ignoring of other people’s religious beliefs and insistence on their own position (Nemati et al., 2017).

All relationships can, in terms of the attachment theory, experience such difficulties in terms of trust, confidence, hope, distance and misunderstandings. This is applicable for the relationship with God, too (Kelley & Chan, 2012; Parenteau et al., 2019). Even when it is theologically and cognitively true that God is always with us (even when a faithful person may have distanced himself from God), the emotional perception that God is distant and not responding may nevertheless be an individual process of pain and grief (Büssing & Dienberg, 2019). In these situations, people require dedicated support—not in the sense of instruction, but understanding and accompaniment. As 24% of the participants stated that they often to regularly have the perception that God has abandoned them completely, and 32% sometimes had this perception, there is a clear demand to care for the people who might be at the crossroads of faith. Nevertheless, 42% stated that they have often to regularly found strategies and resources to cope with these perceptions, while 28% either had not found these strategies at all or had seldom found them. This again underlines that spiritual and/or psychological support is required for the people who do not have access to their resources. According to Musapur et al. (2020), identifying the spiritual and existential transformation of these individuals as a kind of perceptual process to cope with the crisis can help healthcare providers to support them better.

As it has been found in this study, there are some indicators showing who in particular experiences the phases of spiritual dryness. This group includes, namely men and the people who cannot rely on their faith as a resource, those who less often pray, those with low life satisfaction and those who feel lonely and socially isolated because of the pandemic. In many cases, religious people may feel that the current life situation is due to the will of God, as well as other difficult situations. On the other hand, people may also experience that God is not responding as they had expected and feel not “worthy enough” or even abandoned (Büssing et al., 2020b). As a consequence, some may not find inner peace, experience religious struggles, stop praying, etc. (Shirinabadi Farahani et al., 2020).

The positive association between spiritual dryness and taking mood-enhancing medications and meditation practice in this study could be seen in terms of a strategy to cope either with these perceptions and/or with the pandemic-related restrictions. In Iran, many people can buy antidepressants or antianxiety medications from pharmacies without a doctor's prescription, even though it is not legal (Margdari Nejad et al., 2017). The results of a study conducted in Iran demonstrated that perceived stress, physical complaints, anxiety and depression predict sedative use (Gilan et al., 2015). The inability to express emotions may hinder emotional regulation and prohibit successful adjustment, leading to these individuals’ having reduced adjustment capabilities in stressful situations. Perceived stress predicts sedative drug use, possibly because stress and tension produce restlessness and the individual therefore takes drugs to reduce the tension (Gilan et al., 2015). With respect to the frequency of praying (which is negatively related to spiritual dryness because the perceptions that prayers go unanswered are part of the distance perception) it is important to differentiate the frequency of prayers. Interestingly, those who pray at a daily level had the lowest SDS scores and the highest life satisfaction. This indicates that their religious trust and their relation with God seem to be stable which also influences their general life satisfaction. On the other hand, the participants who pray only once a week or once a month had the highest spiritual dryness scores, indicating that their relation with God might be less stable, particularly those who pray at least once per month had the lowest life satisfaction and wellbeing. In Iran, religious practices such as prayers are also among the most important approaches to promoting individual health (Shirinabadi Farahani et al., 2020). However, the frequency of praying is not related to the feeling of being restricted in life, lonely or socially isolated due to the pandemic.

With respect to the predictors of spiritual dryness, regression analyses revealed a complex pattern of triggers, enhancers and putative buffers. The best predictor was taking mood-enhancing medication, probably as an easily accessible strategy to cope with the negative perceptions of spiritual struggles and spiritual exhaustion, which is part of the process. Loneliness (because of the pandemic) and low life satisfaction are relevant predictors that could imply dissatisfaction with the current life and may also affect spiritual wellbeing and relation with God. With the entry of quality of life indicators in the regression model, there was a switch of predictor loading in terms of praying and meditation. Praying was first a negative predictor and meditation a positive one, while praying finally became a positive predictor and meditation a negative one. This might be explained in part by the aforementioned differential usage of praying and meditation intensity. In a study which aimed to investigate the effect of transcendental meditation (as spiritual care) on the spiritual wellbeing of type-2 diabetic amputees, the results confirmed the usefulness of the meditation technique in improving spiritual well-being in the intervention group (Movahed et al., 2020). Moreover, meditation not only may reduce stress (Kemper et al, 2011), but also has positive effects on the quality of life (Dreger et al., 2015), the improvement of spiritual wellbeing (Hartmann et al, 2012) and the increase of hope (Fallah et al., 2011). Accordingly, we have to consider different religious groups (also with respect to their Faith as a strong hold in difficult times) which differ in life satisfaction, too.

However, there is also a positive spiritual development process that should be considered. When the phases of spiritual dryness were overcome, most stated that these feelings inspired them “to help others more” on the one hand and a perception of “greater spiritual serenity and depth” on the other hand. This can indicate a process of spiritual transformation (Büssing et al., 2018b) with both a prosocial and a spiritual direction. According to Tedeschi and Calhoun (1996), although stressful events can result in adverse physical, mental and social effects, the struggle with such events may lead to posttraumatic growth in different aspects of life. Five multidimensional constructs comprise the posttraumatic growth model: personal strength, relating to others, appreciation of life, spiritual changes and new possibilities. However, during the COVID-19 pandemic, these growth processes could be perceived by those who have reliable resources available, but not by all (Büssing et al., 2020c, 2021a, 2021c). As overcoming the phases of spiritual dryness has inspired several individuals to care more for others and to experience a greater spiritual serenity and depth, indicating posttraumatic growth and spiritual transformation despite or because of the pandemic. It is important to provide support in case of crises such as COVID-19 pandemic for all individuals.

## Limitations

The survey was started during the COVID-19 pandemic, which affected the lives of people worldwide, resulting in a decrease of wellbeing and increase of fears and worries. The possibility that this situation has negatively influenced the participants’ perceptions in terms of spiritual dryness cannot be ignored. So far, we have no pre-pandemic data for comparison.

This study was primarily an online survey, and the people without internet access were not able to participate in it. Therefore, it cannot be assumed that our findings are the representative of all Iranian Muslims. Nevertheless, the age range indicates that the elderlies have also participated in the study.

As this study has a cross-sectional design, the direction of causation is of course unclear. For example, the participants may either have experienced the phases of spiritual dryness independently because of the pandemic of or may have perceived the pandemic-related restrictions more strongly because of underlying struggles with God, etc. This can be clarified only in longitudinal studies or qualitative interviews.

## Conclusions

Spirituality and faith are usually reported to be the resources to cope with difficult life situations and may provide meaning in life, hope, inspiration and finding a hold, particularly when one is able to rely on positive religious coping strategies (Thuné-Boyle et al., 2006). However, there are also phases of spiritual dryness, which may challenge religious peoples’ faith and trust in a caring God. This study has found that spiritual dryness is also experienced among Iranian Muslims who participated in this study during the Covid-19 pandemic. The researchers have identified a complex pattern of predictors that may buffer or trigger these perceptions. These perceptions should not be regarded as indicators of “weakness in faith” but as a phase in the lives of devoted religious people. Particularly, the COVID-19 is a challenge that affects the mental stability of people worldwide and is not a matter of surprise that this situation may challenge trust in God, too. Therefore, reliable and humble support of people experiencing these phases is required, as it might be that they are more sensitive toward spiritual struggles than others. Perhaps those who have experienced and overcome these phases have a better understanding that God's ways are different than man’s expectations and are therefore more compassionate toward those who struggle with their faith and with God. At least, this was found in a large fraction of people in this study. Those who respond to these experiences seem to show solidary with those who live far from God. This could be an important task particularly in times of social crisis.

## Data Availability

According to the data protection regulations, the data set cannot be made publicly available. The data can, however, be available upon a reasonable request to the authors.
